# Integrated genomics has identified a new AT/RT-like yet INI1-positive brain tumor subtype among primary pediatric embryonal tumors

**DOI:** 10.1186/s12920-015-0103-3

**Published:** 2015-06-25

**Authors:** Donald Ming-Tak Ho, Chuan-Chi Shih, Muh-Lii Liang, Chan-Yen Tsai, Tsung-Han Hsieh, Chin-Han Tsai, Shih-Chieh Lin, Ting-Yu Chang, Meng-En Chao, Hsei-Wei Wang, Tai-Tong Wong

**Affiliations:** Institute of Microbiology and Immunology, National Yang-Ming University, Taipei, Taiwan; Institute of Clinical Medicine, National Yang-Ming University, Taipei, Taiwan; Genome Research Center, National Yang-Ming University, Taipei, Taiwan; Department of Education and Research, Taipei City Hospital, Taipei, Taiwan; Department of Obstetrics and Gynecology, Hsin-Chu Mackay Memorial Hospital, Hsin Chu, Taiwan; Department of Pathology and Laboratory Medicine, Taipei Veterans General Hospital, Taipei, Taiwan; Division of Pediatric Neurosurgery, Neurological Institute, Taipei Veterans General Hospital, Taipei, Taiwan; School of Medicine, National Yang-Ming University, Taipei, Taiwan; Department of Neurosurgery Taipei Medical University Hospital, Taipei Medical University, Taipei, Taiwan

**Keywords:** Atypical teratoid/rhabdoid tumor, INI1, Pediatric embryonal brain tumor, Transcriptome, Stem cell

## Abstract

**Background:**

Pediatric embryonal brain tumors (PEBTs), which encompass medulloblastoma (MB), primitive neuroectodermal tumor (PNET) and atypical teratoid/rhabdoid tumor (AT/RT), are the second most prevalent pediatric brain tumor type. AT/RT is highly malignant and is often misdiagnosed as MB or PNET. The distinction of AT/RT from PNET/MB is of clinical significance because the survival rate of patients with AT/RT is substantially lower. The diagnosis of AT/RT relies primarily on morphologic assessment and immunohistochemical (IHC) staining for a few known markers such as the lack of INI1 protein expression. However, in our clinical practice we have observed several AT/RT-like tumors, that fulfilled histopathological and all other biomarker criteria for a diagnosis of AT/RT, yet retained INI1 immunoreactivity. Recent studies have also reported preserved INI1 immunoreactivity among certain diagnosed AT/RTs. It is therefore necessary to re-evaluate INI1(+), AT/RT-like cases.

**Method:**

Sanger sequencing, array CGH and mRNA microarray analyses were performed on PEBT samples to investigate their genomic landscapes.

**Results:**

Patients with AT/RT and those with INI(+) AT/RT-like tumors showed a similar survival rate, and global array CGH analysis and *INI1* gene sequencing showed no differential chromosomal aberration markers between INI1(−) AT/RT and INI(+) AT/RT-like cases. We did not misdiagnose MBs or PNETs as AT/RT-like tumors because transcriptome profiling revealed that not only did AT/RT and INI(+) AT/RT-like cases express distinct mRNA and microRNA profiles, their gene expression patterns were different from those of MBs and PNETs. The most similar transcriptome profile to that of AT/RTs was the profile of embryonic stem cells. However; the transcriptome profile of INI1(+) AT/RT-like tumors was more similar to that of somatic neural stem cells, while the profile of MBs was closer to that of fetal brain tissue. Novel biomarkers were identified that can be used to distinguish INI1(−) AT/RTs, INI1(+) AT/RT-like cases and MBs.

**Conclusion:**

Our studies revealed a novel INI1(+) ATRT-like subtype among Taiwanese pediatric patients. New diagnostic biomarkers, as well as new therapeutic tactics, can be developed according to the transcriptome data that were unveiled in this work.

**Electronic supplementary material:**

The online version of this article (doi:10.1186/s12920-015-0103-3) contains supplementary material, which is available to authorized users.

## Background

Pediatric brain tumors are second only to neoplasms of the lymphoid-hematopoietic system in childhood in terms of the number of cases and mortality [1, 2]. Pediatric embryonal brain tumors (PEBTs) are the second most prevalent type of pediatric brain tumors and include medulloblastoma (MB), CNS primitive neuroectodermal tumor (CNS-PNET) and atypical teratoid/rhabdoid tumor (AT/RT). AT/RTs of the central nervous system (CNS) were first described by Rorke *et al.* in 1987. This tumor type was later recognized as a rare, highly malignant entity of CNS embryonal tumors that predominantly occurs in infants and young children with a peak incidence between birth and 3 years of age [3–6]. The distinction of AT/RT from CNS-PNET and MB is of clinical significance because the reported 2-year survival rate of patients with AT/RTs is substantially lower than that of patients with CNS-PNETs or MBs: in Taiwan, the reported 2-year survival rate of patients with AT/RTs is 18 %, while that of patients with a standard-risk MB is 83.9 % [7].

Clinically, cerebellar AT/RT is often misdiagnosed as a PNET or a MB. The diagnosis of AT/RT is based on the presence of rhabdoid tumor cells, which are medium-sized, round-to-oval cells with distinct borders, a large amount of cytoplasm and eccentrically located nuclei. Other morphological features include the presence of small cells with the morphology of PNET cells as well as epithelial and mesenchymal components [5]. AT/RT cells are immunoreactive for a wide range of epithelial, mesenchymal, glial and neural markers, including epithelial membrane antigen (EMA), vimentin (VIM), smooth muscle actin (SMA), and glial fibrillary acidic protein (GFAP) [5, 7, 8]. MB cells are negative by staining for EMA and SMA but are positive for synaptophysin (SYN) [5, 7, 8]. The most widely known tumor suppressor and diagnostic biomarker for AT/RT is INI1 (also known as SMARCB1, hSNF5, BAF47). INI1 belongs to a core member of the ATP-dependent SWI/SNF chromatin-remodeling complex, which is a master regulator of gene expression that is involved in cancer. Genetic studies have shown that deletion or mutation of the *INI1* gene, which is located on 22q11.2, occurs in approximately 75 % [3, 9] to 98 % [10] of AT/RTs. Immunohistochemical (IHC) staining for INI1 is considered a sensitive and highly specific approach for the diagnosis of AT/RT and in the differentiation of this tumor type from PNET and MB [11]. The lack of INI1 protein immunoreactivity has been shown in 100 % [4, 9, 12] to 84 % [13] of AT/RT cases. In contrast, retained INI1 expression (INI1 positive) was noted in all cases of PNETs/MBs [4, 9, 11–13].

With respect to the histopathologic diagnosis of PEBT, in our clinical practice, INI1 as well as EMA, VIM, SMA, GFAP and SYN are included in a panel of IHC markers to distinguish AT/RT from other PEBTs, especially MB. Unexpectedly, we observed several AT/RT-like cases, which fulfilled all other biomarker and histopathologic criteria for a diagnosis of AT/RT, yet still demonstrated INI1 immunoreactivity. It is therefore necessary to understand these atypical cases and to classify them more accurately. For this purpose, we performed a thorough pathological review of our PEBT cases and conducted a systemic genomic analysis. Transcriptomic analysis was also performed using fresh tissues of histopathologically confirmed cases.

## Materials and methods

### Study materials and clinical data

Study cases were retrieved from the surgical pathology files of the Department of Pathology and Laboratory Medicine, Taipei Veterans General Hospital (VGH-TPE), Taiwan. The Parent/legal guardian of patients in this study provided informed consent, and all procedures were approved by the Institutional Review Board of VGH-TPE (VGHIRB No.:2011-11-007GA & 2011-11-008GA). Fresh tumor tissues that were removed during surgery were snap-frozen and stored in liquid nitrogen until DNA and RNA extraction. The overall survival time was calculated as the time from surgery until death or the time from surgery until the last follow-up appointment for the patients who survived. A Mann–Whitney test was used to compare age differences among the different groups of patients. The differences in survival times were assessed with the log-rank test.

### Histopathologic diagnosis of AT/RT and MB

A diagnosis of AT/RT was based on the morphologic features of the tumor and the results of IHC, as described in our previous reports [7, 14]. With regard to the morphologic features, rhabdoid cells, which were either large, pale, bland cells or the classical type similar to those observed in malignant rhabdoid tumors of the kidney, were essential for diagnosis [8, 15]. Other features, such as cells that are similar to primitive neuroectodermal cells, an epithelial component and a mesenchymal component, could also be observed. An IHC diagnostic panel included the following markers: epithelial membrane antigen [EMA; monoclonal, dilution 1:40, Dako, Glostrup, Denmark, Histostain SP Broad Spectrum (HRP), Zymed Lab., Carlsbad, USA (Histostain); antigen retrieval using a microwave, three cycles for 5 min each (M)], vimentin (VIM; monoclonal, 1:600, Dako, Histostain, M), smooth muscle actin (SMA, HHF-35; monoclonal, 1:75, Dako, Carpinteria, CA, USA, Histostain, M), and glial fibrillary acidic protein (GFAP; monoclonal, 1:300, Dako, Histostain, M) [5, 8, 14]. The rhabdoid cells were immunoreactive for two or more of the above-listed antibodies. IHC for INI1 (anti-BAF47; monoclonal, 1:40, BD Transduction Laboratories, San Diego, CA, USA, Histostain, M) and SMARCA4 (anti-BRG1; monoclonal, 1:100, Abcam, Cambridge, U.K., Histostain, M) was included in all cases in this study. With regard to a diagnosis of MB, besides the presence of the known morphology of this tumor type, the features of AT/RT as listed above were absent. Immunostaining for synaptophysin (SYN; monoclonal, 1:50, Novocastra, Newcastle upon Tyne, U.K., Histostain, M) was included to confirm the diagnosis and to distinguish it from AT/RT; IHC for SYN was also performed in all AT/RT cases. Positive and negative controls were included with each batch of sections to confirm the consistency of the analysis in all of the stains that were performed in this study. As for the INI1 staining, positive control consisted of endothelial cells within the tumor. Negative controls consisted of staining without applying the primary antibody and staining of a known INI1 negative AT/RT.

### Direct sequencing, reverse transcription-PCR (RT-PCR) and Quantitative real-time reverse transcription-PCR (qRT-PCR)

Genomic DNAs and total RNA were isolated from fresh-frozen tumor samples by the DNeasy Blood& Tissue Kit and RNAeasy (Qiagen) according to the manufacturer’s instruction (Qiagen, GmbH, Germany), respectively. Genomic DNAs were used to peform PCR using specific INI1 gene primers and then sequenced by direct sequencing. For RT-PCR and qRT-PCR, 1 μg of total RNA was used to perform reverse transcription (RT) using the RevertAid™ Reverse transcriptase kit (Cat. K1622; Fermentas, Glen Burnie, Maryland, USA) as directed by the manufacturer. For INI1 RT-PCR, a paired-primer encompassing exon 5 and 6 was used. For INI1, the forward primer was 5′-AACAGGAACCGCATGGGCCG-3′, and the reverse primer was 5′-GCCCGTGTTCCGGATGGCAA-3′ (amplicon size: 579 bps). For GAPDH, the forward primer was 5′-CAAGGTCATCCATGACAACTTTG-3′, and the reverse primer was 5′-GTCCACCACCCTGTTGCTGTAG-3′ (amplicon size, 496 bps). Quantitative real-time PCR reactions were performed using Maxima™ SYBR Green qPCR Master Mix (Cat. K0222; Fermentas, Glen Burnie, Maryland, USA), and the specific products were detected and analyzed using the StepOne™ sequence detector (Applied Biosystems, USA). The expression level of each gene was normalized to GAPDH expression. For GAPDH, the forward primer was 5′-CCAGCCGAGCCACATCGCTC-3′ and the reverse primer was 5′-ATGAGCCCCAGCCTTCTCCAT-3′. For SOX4, the forward primer was 5′- TCGCTGTACAAGGCGCGGAC-3′ and the reverse primer was 5′-TTCTCCGCCAGGTGCTTGCC-3′. For ERBB2, the forward primer was 5′- AGTACCTGGGTCTGGACGTG-3′ and the reverse primer was 5′-CTGGGAACTCAAGCAGGAAG-3′. For OLIG2, the forward primer was 5′-CAGAAGCGCTGATGGTCATA-3′ and the reverse primer was 5′-TCGGCAGTTTTGGGTTATTC-3′.

### Array CGH (aCGH) analysis

As described in our previous study [14], the samples were mixed with control DNA samples from healthy donors before they were subjected to the analysis. A Human Genome CGH Microarray Kit 244A (Agilent Technologies, USA) with 99,000 probes and an average probe spatial resolution of 15.0 kb was used. aCGH was performed according to the protocol suggested by Agilent. Data analysis was performed using CGH Analytics 3.4 (Agilent Technologies) using the default parameters. Briefly, chromosomal abbreviations were calculated using the ADM2 statistic algorithm with a moving average window of 1 Mb; additionally, the default thresholds of ADM2 recommended by Agilent were used to make an amplification or deletion call.

### Gene expression microarray (GEM) and computational analyses

Array data on adult neural stem cells and embryonic stem cells were obtained in our previous study [16] and from the Gene Expression Omnibus (GEO; http://www.ncbi.nlm.nih.gov/geo/) dataset GSE9940. An mRNA expression array analysis was performed as previously described [16, 17]. Briefly, an Affymetrix™ HG-U133 Plus 2.0 whole genome array was used. RMA log expression units were calculated from the Affymetrix GeneChip array data with the ‘affy’ package of the Bioconductor (http://www.bioconductor.org/) suite software for the R statistical programming language (http://www.r-project.org/). The default RMA settings were used to background correct, normalize and summarize all expression values. Significant differences between sample groups were identified by the ‘limma’ package [16]. Briefly, a t-statistic was calculated as normal for each gene, and a p-value was then calculated with a modified permutation test [16]. To control for the multiple testing errors, a false discovery rate (FDR) algorithm was then applied to these p-values to calculate a set of q-values: thresholds of the expected proportion of false positives or false rejections of the null hypothesis. Heat maps were then created by dChip software (http://www.dchip.org/). Classical multidimensional scaling (MDS) was performed with the standard function of the R program to provide a visual impression of how the various sample groups are related. Gene annotation was performed by the ArrayFusion web tool (http://microarray.ym.edu.tw/tools/arrayfusion/) [18]. Principal component analysis (PCA) was performed with Partek Genomics Suite software (http://www.partek.com) to provide a visual impression of how the various sample groups are related. All array data have been submitted to the NCBI Gene Expression Omnibus (GEO) database, and the accession number is GSE65132 (Additional file 2).

### MicroRNA microarray analysis

The Agilent Human miRNA Microarray Kit V2 (Agilent, Foster City, CA, USA) containing probes for 723 human microRNAs from the Sanger database v10.1 was used. GeneSpring GX 9 software (Agilent, USA) was used for value extraction. A 2-tailed Student’s *t*-test was then used for the calculation of the p value for each miRNA probe.

## Results

### Clinical features of the included primary pediatric embryonal brain tumors

**Fig. 1:**
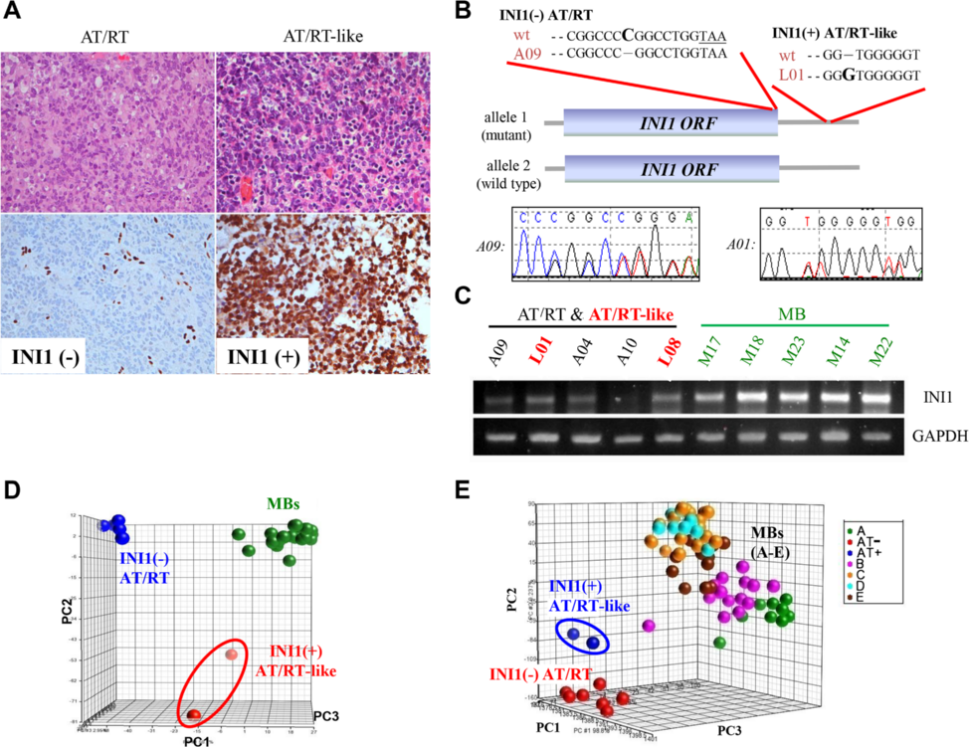
Identification of INI1(+) AT/RT-like cases among Taiwanese pediatric embryonal brain tumor cases. (**a**) Hematoxylin and eosin stain and INI1 stain of pediatric AT/RT and AT/RT-like cases, one with negative INI1 immunoreactivity (left) and one with positive INI1 immunoreactivity (right; anti-INI1, 400x). (**b**) Schematic representation of the results of *INI1* gene sequencing. Patient A09 has only one mutated allele in the AT/RT: a single C deletion (in bold) was detected just before the INI1 stop codon (TAA; underlined). This resulted in a frame-shift mutation. In patient L01 with an INI1(+) AT/RT-like tumor, a single G insertion (in bold) was detected in the 3′UTR region of one *INI1* allele. No protein abrogation was expected in this case. ORF indicates the open reading frame. (**c**) An examination of INI1 mRNA expression in tumor tissues by RT-PCR. (**d**) A PCA plot was drawn according to mRNAs that are differentially expressed between MB, AT/RT, and AT/RT-like cases (q < 10^−5^). INI1(+) AT/RT-like tumors have distinct mRNA expression profiles similar to those of AT/RTs and MBs. (**e**) Gene expression analysis of Taiwanese INI1(+) AT/RT-like cases and another published Caucasian MB data set. A PCA plot drawn according to the whole transcriptome again showed that INI1(+) AT/RT-like tumors were different from MBs and INI1(−) AT/RTs (A: Wnt subgroup; B: SHH subgroup; C: subgroup which expressed neuronal differentiation characteristic; D: subgroup which expressed neuronal and photoreceptor characteristic; E: subgroup which expressed photoreceptor characteristic and increased protein biosynthesis/cell cycle)

The diagnosis of AT/RTs and other PEBTs, especially MBs, was based on the morphologic and IHC features described in our previous reports (Additional file 1-A) [7, 14]. Fresh tissues from 45 patients with PEBT (9 INI1- AT/RT, 5 INI1+ AT/RT-like, and 31 INI1+ MB) were used in the genomics studies (Table 1). With regard to IHC assays, EMA, VIM, SMA, GFAP and INI1 were used as diagnostic markers, and all AT/RT or AT/RT-like cases demonstrated positivity for at least two of these markers (Table 2). The locations within the CNS of the AT/RT and INI1+ AT/RT-like tumors included the cerebellum and the lateral ventricle. Pediatric cerebellar MB cases were also collected as control samples, and all of those tumors were INI1+ (Table 1). Examples of loss (INI1-) and preservation (INI1+) of INI1 expression according to IHC in AT/RTs and AT/RT-like cases are shown in Fig. 1a and are as described in our previous work [14]. The IHC results were clear because the tumor cells were either diffusely positive or diffusely negative (Fig. 1a). Mutations in the SMARCA4 subunit are considered alternative mutations that may be present in AT/RT-like tumors [19, 20]; however, by IHC, we found no SMARCA4 loss in our AT/RTs and AT/RT-like tumors (Table 1 and Additional file 1-B).

**Table 1:** Clinical details and INI1 IHC data of PEBT cases enrolled in aCGH and transcriptome studies

Case	Age	Sex	Site	Histo.	INI1	SMARCA4	aCGH	mRNA	microRNA
No.	(yr)			Dx	IHC	IHC		array	array
L01	8.3	m	cblm	AT/RT-like	+	+	+	+	+
L02	4.2	m	cblm	AT/RT-like	+	+	+	ND	ND
L06	3.5	f	cblm	AT/RT-like	+	+	+	ND	ND
L07	9.8	m	cblm	AT/RT-like	+	+	+	ND	ND
L08	9.4	f	cblm	AT/RT-like	+	+	+	+	+
A03	2.3	f	cblm	AT/RT	-	+	+	ND	ND
A04	4.5	f	lat. vent.	AT/RT	-	+	+	+	+
A05	0.1	m	cblm	AT/RT	-	+	+	ND	ND
A09	5.2	f	cblm	AT/RT	-	+	+	+	+
A10	1.6	m	cblm	AT/RT	-	+	ND	+	+
A11	1.4	f	cblm	AT/RT	-	+	ND	+	ND
A12	0.6	f	cblm	AT/RT	-	+	ND	+	ND
A13	5	m	cblm	AT/RT	-	+	ND	+	ND
A14	1.6	m	cblm	AT/RT	-	+	ND	+	ND
M01	8.7	m	cblm	MB, cl	+	+	+	ND	ND
M02	9.2	f	cblm	MB, an	+	+	+	ND	ND
M03	4	m	cblm	MB, cl	+	+	+	ND	ND
M04	6.3	m	cblm	MB, ds	+	+	+	ND	ND
M05	3.5	f	cblm	MB, ds	+	+	+	ND	ND
M06	9.4	m	cblm	MB, cl	+	+	+	ND	ND
M07	7.6	m	cblm	MB, an	+	+	+	ND	ND
M08	14.3	f	cblm	MB, cl	+	+	+	ND	ND
M09	13.6	m	cblm	MB, cl	+	+	+	+	ND
M10	12.2	f	cblm	MB, cl	+	+	+	ND	ND
M11	4.1	f	cblm	MB, cl	+	+	+	+	ND
M12	4.3	m	cblm	MB, cl	+	+	ND	+	ND
M13	3.2	m	cblm	MB, cl	+	+	ND	+	+
M14	3	m	cblm	MB, cl	+	+	ND	+	+
M15	1.5	f	cblm	MB, an	+	+	ND	+	+
M16	9.1	f	cblm	MB, an	+	+	ND	+	+
M17	13	f	cblm	MB (*)	+	+	ND	+	+
M18	18.2	m	cblm	MB, cl	+	+	ND	+	+
M19	5.8	f	cblm	MB, cl	+	+	ND	+	+
M20	5.1	m	cblm	MB, cl	+	+	ND	+	+
M21	3	m	cblm	MB, cl	+	+	ND	+	+
M22	8.5	f	cblm	MB, an	+	+	ND	+	+
M23	2.2	m	cblm	MB, ds	+	+	ND	+	+
M24	5.8	f	cblm	MB (*)	+	+	ND	+	ND
M25	7.6	f	cblm	MB, cl	+	+	ND	+	+
M26	12.2	m	cblm	MB, an	+	+	ND	+	ND
M27	6.5	f	cblm	MB, an	+	+	ND	+	ND
M28	2.1	m	cblm	MB, ds	+	+	ND	+	ND
M29	6.3	m	cblm	MB, cl	+	+	ND	+	ND
M30	10	m	cblm	MB, an	+	+	ND	+	ND

Site: cblm: cerebellum, lat.vent: lateral ventricle

Histo. Dx. (Histopathological diagnosis): cl: classic, ds: demoplastic, an: anaplastic

INI1 IHC: −: loss of INI1 expression (INI1-), +: retained INI1 expression (INI1+);

SMARCA4 IHC: −: loss of SMARCA4 expression, +: retained SMARCA4 expression;

aCGH & GEM: +:performed, ND: not determined

(*)M17 with focal anaplasia; M24 with myogenic and melanocytic differentiation

**Table 2:** IHC features of AT/RT and AT/RT-like cases enrolled

Case no.	INI1	EMA	VIM	SMA	GFAP
L01	**+**	**-**	**+**	**+**	**+**
L02	**+**	**-**	**+**	**+**	**-**
L06	**+**	**+**	**+**	**+**	**+**
L07	**+**	**+**	**-**	**+**	**+**
L08	**+**	**+**	**+**	**+**	**-**
A03	**-**	**+**	**+**	**+**	**+**
A04	**-**	**+**	**+**	**+**	**+**
A05	**-**	**+**	**+**	**-**	**+**
A09	**-**	**+**	**+**	**+**	**-**
A10	**-**	**+**	**+**	**+**	**+**
A11	**-**	**+**	**+**	**+**	**+**
A12	**-**	**+**	**+**	**+**	**+**
A13	**-**	**+**	**+**	**+**	**+**
A14	**-**	**+**	**+**	**+**	**+**

### Similar genomic DNA aberrations in INI1 (−) AT/RTs and INI1(+) AT/RT-like cases

AT/RT cases A03-05 and A09-10, which were all INI1(−), were used in our previous study [14]. By direct sequencing, we determined that the *INI1* gene region in these 5 cases was intact, while the expression of the INI1 protein in these cases was paradoxically lost. A novel yet unidentified posttranscriptional regulatory mechanism that occurs as part of INI1 protein synthesis likely exists in AT/RT tumor cells [14]. We conducted a similar analysis of the genomic DNA from cases in our current series, which included 5 AT/RT-like tumors (L01-L02 & L06-L08, Table 1) and an additional 4 new AT/RT cases (A11-A14, Table 1). PCR-amplified genomic DNA samples that were isolated from fresh-frozen tumors were used for direct *INI1* gene sequencing, and mutations were found in only two cases. L01 (INI1+) showed a G insertion in exon 9 in one allele. Because this insertion was located in the 3′-UTR region of the *INI1* mRNA, no abrogation of the protein would be expected (Fig. 1b, inserted G in bold). With regard to the AT/RTs, only one case (A09), which was documented in our previous report [14], acquired a C deletion in exon 9 in one allele (Fig. 1b). Such a deletion would lead to a frame-shift mutation and would generate a new INI1 protein with an extra 100-aa tail (Fig. 1b, deleted C in bold and TAA stop codon underlined). Thus, no differences in the *INI1* gene region were detected between the INI1(+) AT/RT-like and INI1(−) AT/RT populations. RT-PCR experiments confirmed the expression of INI1 transcripts in both INI1(+) AT/RT-like cases (L01 and L08, Fig. 1c) and INI1(−) AT/RTs [14] (A04 and A09, Fig. 1c).

Deletion of the *INI1* gene, which is located at 22q11.2, has been proposed to be a unique feature of AT/RT tumors [3, 9]. However, our previous array CGH analysis on Taiwanese INI1(−) AT/RT cases showed no deletion around the *INI1* gene (Table 3) [14]. We extended the aCGH analysis to INI1(+) AT/RT-like cases, and still no 22q11.2 deletion could be found (Table 3). The INI1(+) and INI1(−) cases shared common chromosomal aberrations, including gains in 1q21.31 (150836484 ~ 150848568), 11p15.5 ~ p15.4 (274838 ~ 2917590), 11q11 (55129329 ~ 55195049), 14q32.33 (105602756 ~ 105630148), and 22q13.31 (44813553 ~ 44849176) (Table 3). The areas that were most commonly deleted were 7q36.1 (151592619 ~ 151887233), 8p11.23 (39356595 ~ 39482044), 12p13.31 (7696275 ~ 8068377), 15q11.2 (18810004 ~ 19910926) and 21p11.1 (10117898 ~ 10144936) (Table 3). Chromosomal aberrations in MB, including the well-known iso-17q mutation (17p11.2 ~ q25.3, 20851757 ~ 78653717 [14], were not detected in any of INI1(+) AT/RT-like or INI(−) AT/RT cases, which indicates that no patients with MB were misdiagnosed or mistaken to have INI1(+) AT/RT-like tumors.

**Table 3:** Array CGH analysis showed no differential chromosomal aberration between INI1(−) AT/RTs & INI1(+) AT/RT-like cases

		Array CGH results	
Case No.	INI1	Gain	loss
L01*	+	*1q21.3, 11q11, 14q32.33,*	*8p11.23*
L02	+	*1q21.3, 14q32.33, 22q11.23*	*7q36.1, 12p13.31, 21p11.1*
L06	+	*1q21.3, 11q11, 22q11.23, 22q13.31*	*7q36.1, 12p13.31, 15q11.2, 21p11.1*
L07	+	*11p15.5 ~ p15.4, 14q32.33, 22q11.23, 22q13.31*	*7q36.1, 8p11.23, 12p13.31, 21p11.1*
L08	+	*11p15.5 ~ p15.4, 11q11, 14q32.33, 22q11.23, 22q13.31*	*15q11.2, 21p11.1*
A03	-	*1q21.3, 11p15.5 ~ p15.4, 11q11, 14q32.33, 22q11.23*	*8p11.23, 15q11.2*
A04	-	*11p15.5 ~ p15.4, 14q32.33, 22q13.31*	*7q36.1, 8p11.23, 21p11.1*
A05	-		*15q11.2, 21p11.1*
A09**	-	*14q32.33, 22q11.23*	*7q36.1, 12p13.31*

* Mutation on 1 allele; no mutation on the protein level

** Mutation on 1 allele; results in a new protein with additional 79 a.a

### Distinct gene expression patterns among different subgroups of PEBTs

To further verify that INI1(+) AT/RT-like cases were not misdiagnosed MBs, as well as to provide new insights into the molecular differences among MB, AT/RT, and INI1(+) AT/RT-like cases, we examined the transcriptome patterns of these 3 closely related embryonal brain tumors by microarray analysis. A principle component analysis (PCA) plot based on genes that differentiate MB and AT/RTs and AT/RT-like cases (with a positive false discovery rate (pFDR) threshold of q < 10^−5^, 496 probe sets) showed that the AT/RT and MB cases we diagnosed were distinct from each other at the mRNA level (Fig. 1d, Additional file 2). Furthermore, the genetic profiles of AT/RTs and INI1(+) AT/RT-like tumors were distinct (Fig. 1d).

With another independent MB data set published by Kool *et al.* [21] (GEO database accession No. GSE10327) as an independent cohort, we still observed distinct transcriptome patterns among MBs, AT/RTs and INI1(+) AT/RT-like tumors (Fig. 1e). We also compared the transcriptomes of AT/RTs and INI1(+) AT/RT-like tumors to those of PNETs. An MDS plot showed that the transcriptomes of either AT/RTs or INI1(+) AT/RT-like tumors were significantly different from those of PNETs (Additional file 1-C), which indicates that the INI1(+) AT/RT-like cases in our series were not misdiagnosed PNETs.

### Stem cell traits of different groups of embryonal brain tumors reflect their distinct clinical prognoses

**Fig. 2:**
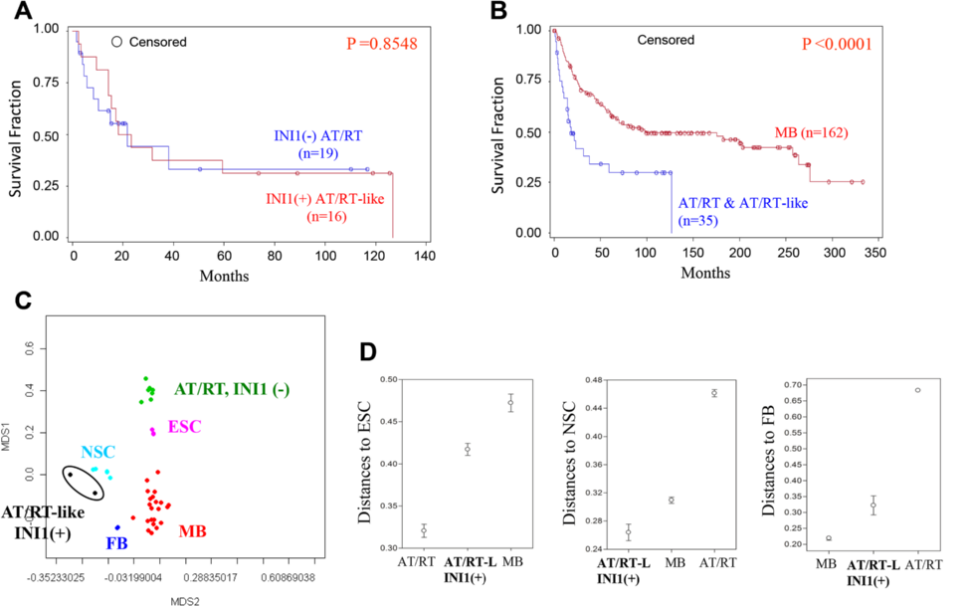
Gene signatures reflect the clinical prognostic status of different subtypes of embryonal brain tumors. (**a**) Overall survival rates of the INI1(−) AT/RTs (n = 19) and INI1(+) AT/RT-like (n = 16) cases included in the IHC analysis. (**b**) Survival curves of 199 cases of primary pediatric CNS embryonal tumors (162 MBs, 19 AT/RTs, and 16 INI1(+) AT/RT-like cases) that were included in the IHC validation study. All patients with AT/RTs or AT/RT-like tumors had a worse overall survival than patients with MBs (P < 0.0001). (**c**) An MDS plot based on genes that are differentially expressed in the 3 subtypes of embryonal brain tumors (q < 0.001) show the relationships among AT/RTs, MBs, INI1(+) AT/RT-like tumors and different stem or progenitor cells. ESC: embryonic stem cell; NSC: adult neural stem cell; FB: fetal brain tissues. (**d**) Analysis of the transcriptome distance between AT/RTs, MBs, INI1(+) AT/RT-like tumors and different stem or progenitor cells. AT/RT-L: AT/RT-like

To evaluate the survival outcomes of patients with AT/RTs and INI1(+) AT/RT-like tumors, we expanded the case numbers in the Kaplan-Meier (KM) estimator analysis. KM analysis and a log-rank test revealed that patients with INI1(−) AT/RTs and INI1(+) AT/RT-like tumors had a similar overall survival rate (19 AT/RTs and 16 AT/RT-like cases; Fig. 2a). In contrast, all 35 patients with AT/RTs or AT/RT-like tumors had a worse overall survival than patients with MBs (P < 0.0001; Fig. 2b). It is clear that cancer cells possess traits that are reminiscent of those ascribed to normal stem cells and that the degree of stem cell gene expression correlates with patient prognosis. Histologically poorly differentiated tumors or late-stage tumors show preferential overexpression of genes that are normally enriched in ES cells (ESCs) or in somatic stem cells, which may partly explain why these tumors are more malignant [22, 23]. We hypothesized that the distinct clinical survival trends of different PEBTs might be reflected by their gene expression traits. The array data of pluripotent ESCs, multipotent somatic neural stem cells (NSCs) and terminally differentiated fetal brain (FB) cells were included in a comparative transcriptome analysis. As expected, MBs were more similar to FB tissue (Fig. 2c). The transcriptome profiles of INI1(−) AT/RTs and INI1(+) ATRT-like tumors were most similar to those of ESCs and NSCs, respectively (Fig. 2c). Such impressions were quantified by the measurement of the average linkage distances between different embryonal brain tumors with respect to ESCs, to NSCs, or to FB cells (Fig. 2d).

### Novel biomarkers for MBs, INI1(−) AT/RTs, and INI1(+) AT/RT-like tumors

**Fig. 3:**
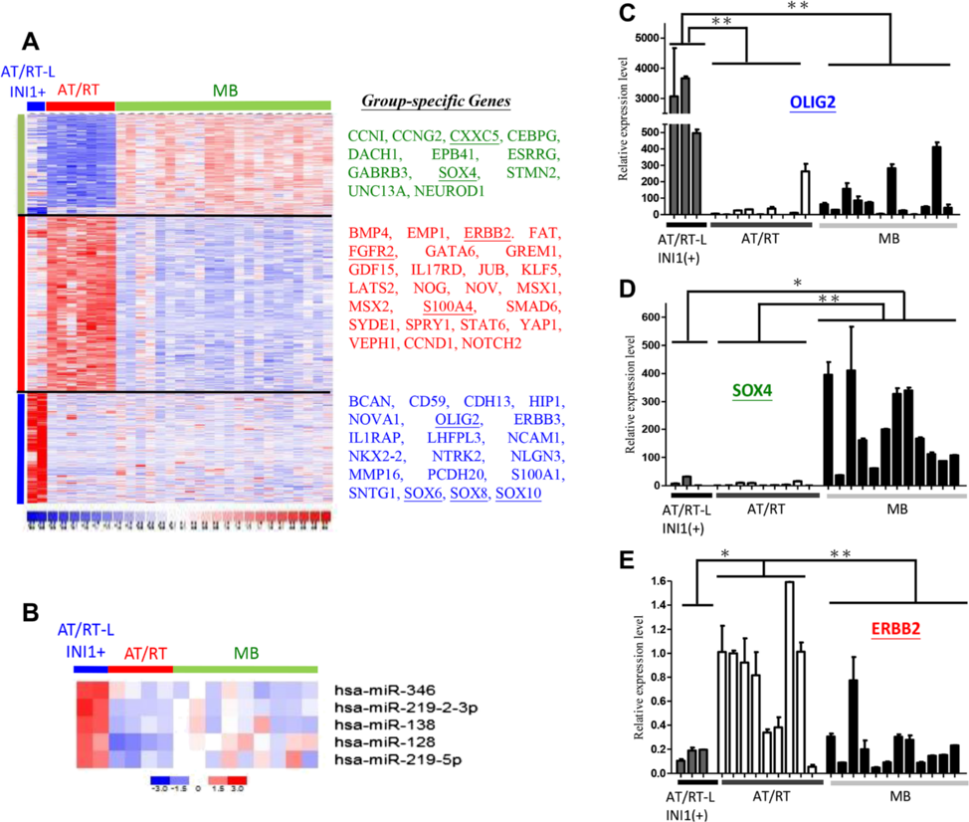
Genes that distinguish AT/RTs, MBs, and INI1(+) AT/RT-like tumors. (**a-b**) Heat maps show group-specific genes (***a***) and microRNAs (***b***). AT/RT-L: AT/RT-like. Red: up-regulation, Blue: down-regulation. (**c-e**) Real-time PCR validation of OLIG2 (***c***), SOX4 (*D*) and ERBB2 (***e***) array data on new batches of patient RNAs. ^*^ *P* < 0.05; ^**^ *P* < 0.01

To identify novel biomarkers and the pathogenetic mechanisms of these 3 subtypes of embryonal brain tumors, genes that are differentially expressed between each tumor type were also identified. A gene expression heat map for these genes indicated their unique expression patterns in these tumor subtypes (Fig. 3a). The FGFR2 growth factor receptor, the S100A4 stemness gene and the ERBB2 (HER2/neu) oncogene were dominant in the INI1(−) AT/RT cases (Fig. 3a, underlined). CXXC5 and SOX4 were enriched in MBs, while OLIG2, SOX6, SOX8, and SOX10 were expressed in 2 INI1(+) AT/RT-like samples (Fig. 3a). We also profiled the microRNA patterns in INI1(+) AT/RT-like samples, INI1(−) AT/RTs and MBs. Five microRNAs (miR-128, −138, −219-5p, −219-2-3p and −346) were found to be abundantly expressed in INI1(+) AT/RT-like cases (Fig. 3b).

Array data on OLIG2, SOX4 and ERBB2 were validated by quantitative PCR (qPCR) (Fig. 3c-e, Additional file 2). OLIG2 was shown to be significantly overexpressed in INI1(+) AT/RT-like tumors (Fig. 3c), while SOX4 was overexpressed in MBs (Fig. 3e). Such a difference in the expression levels of these genes can be used to distinguish MBs from AT/RTs and, more critically, can be used to distinguish AT/RTs from INI1(+) AT/RT-like tumors.

## Discussion

CNS AT/RTs are a specific entity of pediatric embryonic tumors. The ability to distinguish AT/RT from MB is of clinical significance because the reported survival rate of patients with AT/RTs is significantly lower than that of patients with a standard-risk MB [3]. The dismal outcome of patients with AT/RT is due to their poor response to conventional adjuvant therapy protocols, but recent studies have shown that improvements in patient survival can be achieved with intensive aggressive therapy or radiotherapy as an initial treatment [6, 24, 25]. Here, we report the identification of a new subtype of AT/RT-like tumors among cases of primary pediatric embryonal brain tumors in Taiwan and our data support the reliability of our original pathological diagnoses.

The INI1-positive AT/RT-like subtype may not be restricted to cases of PEBT in this Taiwanese population. A recent case report from Italy illustrates an INI1(+) AT/RT case in a 9-month-old boy whose tumor showed retained INI1/SMARCB1 expression by IHC and lacked genetic alterations in the *INI1* gene [19]. Instead, the tumor had a nonsense mutation and loss of protein expression of another member of the SWI/SNF chromatin-remodeling complex, the ATPase subunit SMARCA4 (BRG1) due to a homozygous SMARCA4 mutation [c.2032C > T (p.Q678X)] [19]. Preserved INI1 protein expression and a SMARCA4 mutation were also observed in familial rhabdoid tumor predisposition syndrome (RTPS), which has been linked to heterozygous SMARCB1 germline mutations. Two sisters with RTPS whose tumors lacked mutations in INI1/SMARCB1 were diagnosed in Germany [20]; instead of a mutation in INI1/SMARCB1, mutations were again found in SMARCA4 in both patients. This result implied that SMARCA4 is a second member of the SWI/SNF complex involved in cancer predisposition and that SMARCA4 may play an essential role in the pathogenesis of new INI1/SMARCB1-positive AT/RT-like tumors. Whether SMARCA4 or other proteins within the SWI/SNF chromatin-remodeling complex are also involved in the pathogenesis of these Taiwanese INI1(+) AT/RT-like cases is currently under investigation.

Based on array CGH analysis, the abnormalities in the genomic DNA of the AT/RTs and the AT/RT-like cases we studied were indistinguishable yet distinct from those of published MB cases [14]. The *INI1* region is intact in both INI1(+) AT/RT-like and INI1(−) AT/RT cases (Fig. 1b-c). Genetic studies of western cohorts have shown that deletion or mutation of the *INI1* gene occurs in approximately 75 % [3, 9] to 98 % [10] of the AT/RTs studied. Nevertheless, in our previous study, we found no deletion around the *INI1* gene in Taiwanese patients [14]. Subgroups of patients with AT/RTs have tumors that express *INI1* mRNA, even though the tumors in our series were negative for INI1 protein by IHC [14]. Our study reveals that a novel yet unidentified posttranscriptional regulatory mechanism(s) that occurs after INI1 protein synthesis exists in AT/RT tumor cells. The application of *INI1* genomic DNA as an essential diagnostic tool is therefore not invariably suitable, at least in Taiwanese cases.

Tumor development, progression, and prognosis remain positioned at the front line of medical research. Clinically, cancer cells with a poor differentiated pathological grade usually have a worse therapeutic response [22]. It has been demonstrated that cMyc, but not other tested oncogenes, is sufficient to reactivate the ESC-like program in normal and cancer cells [26]. The convergence of dedifferentiation and cancer malignancy also comes from the discovery that the epithelial-mesenchymal transition (EMT), which is a critical mechanism that mediates embryogenesis and cancer metastasis, can induce the formation of cancer spheres from transformed breast cancer or colon cancer epithelial cells. This concept is also derived from the discovery that tumor stem cells that have undergone EMT are more motile and show greater metastatic ability [27–29]. Among the three subtypes of PEBTs that we analyzed, we also discovered distinct stem/progenitor cell signatures in these tumors as well as expression levels of stemness genes that correlated well with patient prognosis (Fig. 2). Genes that are responsible for tumor stemness and resistance to therapy remain to be elucidated and require further investigation.

We uncovered unique gene expression patterns among AT/RTs, AT/RT-like tumors and MBs, and identified OLIG2 as a potential new biomarker for AT/RT-like cases (Fig. 3c). OLIG2 is expressed in neural progenitor cells [30], which corresponds with the finding that INI1(+) ATRT-like tumors shared a closest transcriptome profile to that of NSCs (Fig. 2c-d). OLIG2 also showed in subgroup of CNS-PNET [31], but according to our PCA plot (Additional file 1-C), they express distinct transcriptome. Therefore, INI1 plus OLIG2 and other existed biomarkers INI1 plus OLIG2 immunostains may help to accurately diagnose this new AT/RT-like subtype among cases of primary PEBTs. In addition, some genes were down-regulated in AT/RT-like tumors, such as SRD5A1 and CREB3L4. These genes have a chance to be biomarkers in AT/RT-like tumors, but it still need more samples to verify.

SOX4 is a potential new biomarker for MBs. Sox4 and Sox11, two HMG-box transcription factors, play central regulatory roles during neuronal maturation [32]. Other transcriptome analyses based on microarray or suppression subtraction hybridization also identified SOX4 as an abundant protein in human MBs [33, 34]. IHC studies have verified that the Sox4 and Sox11 proteins are strongly expressed in most classical MBs [34, 35]. Our array and RT-qPCR series contain classical MBs, desmoplastic MBs and anaplastic MBs, and all cases of MB expressed abundant SOX4 transcripts compared with cases of INI1(+) AT/RT-like cases or INI1(−) AT/RTs.

ERBB2 (HER2/neu) was found to be overexpressed in the AT/RT cases that we profiled. The ERBB2/HER2/neu oncogene, the human epidermal growth factor receptor 2, is a member of the epidermal growth factor receptor (EGFR/ErbB) family. The amplification or over-expression of this gene has been shown to play an important role in the pathogenesis and progression of certain aggressive types of breast cancer and advanced endometrioid carcinoma [23]. In recent years, ERBB2 has evolved to become an important prognostic biomarker and therapeutic target for breast cancer, and targeted therapies such as Herceptin™ (trastuzumab) and Omnitarg™ (pertuzumab) are now available clinically. The possibility that existing clinical drugs can be repurposed for the treatment of AT/RTs has yet to be evaluated.

In addition, we also find 5 miRNAs were up-regulated in AT/RT-like tumors. MiR-138 have been reported to serve as tumor suppressor in glioblastoma multiforme [36] or oncomiR in malignant glioma [37], respectively. MiR-128 and miR-219-5p are considered to be tumor suppressors, since which are found to inhibit proliferation, migration and progression in GBM [38] and gliomas [39]. Until now, miR-346 and miR-219-2-3p don’t publish in other brain tumor types. Therefore, miR-346 and miR-219-2-3p have a chance to be specific biomarkers for AT/RT-like tumors.

## Conclusions

In conclusion, IHC and transcriptome studies of our primary embryonal brain tumor series identified a novel INI1 (+) AT/RT-like subtype among Taiwanese pediatric patients. Distinct gene expression patterns of closely related embryonal tumors were also provided. Careful diagnosis and clinical care of patients with different subtypes of embryonal brain tumors will benefit daily clinical practice. In addition, INI1(+) AT/RT-like cases are rare and hard to collect genetic data. Therefore, a collaborative international effort needs to happen immediately, increasing tumor numbers will help us to realize and supply specific biomarkers to distinguish between INI1(+) AT/RT-like tumors and other pediatric embryonal tumors.

## Additional files

Additional file 1:
**(A) IHC features used for the diagnosis of AT/RT and MB in our clinical practice for pediatric embryonal brain tumors.** (**B**) IHC results for SMARCA4 in AT/RT-like, AT/RT and MB samples. (**C**) Both INI1(+) AT/RT-like tumor and INI(−) AT/RT possess distinct mRNA profiles compared with those of PNETs. MDS plots were drawn based on genes that can differentiate AT/RT from AT/RT-like tumors (with a positive false discovery rate (pFDR) threshold of q < 0.001) show the relationships among AT/RTs, PNETs, and INI1(+) AT/RT-like tumors. Additional file 2:
**Expression data from AT/RTs, AT/RT-like tumors and medulloblastomas.**
