# Supplementary figures and images for: Integrated genomics has identified a new AT/RT-like yet INI1-positive brain tumor subtype among primary pediatric embryonal tumors
Source: BMC Med Genomics. 2015 Jun 25;8:32. doi: 10.1186/s12920-015-0103-3 (PMC4480900; doi:10.1186/s12920-015-0103-3)

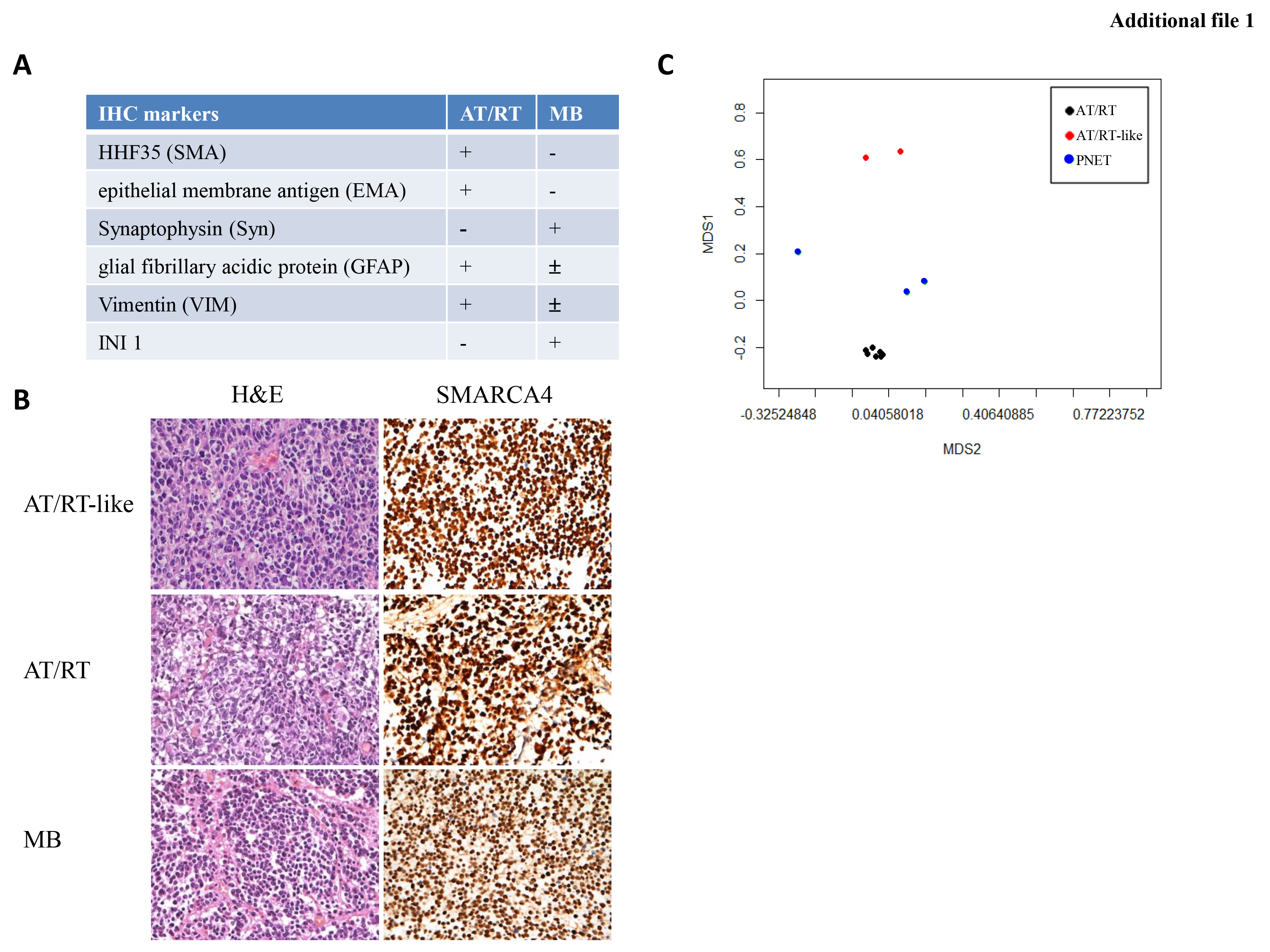

Supplement: Additional file 1: — (A) IHC features used for the diagnosis of AT/RT and MB in our clinical practice for pediatric embryonal brain tumors. (B) IHC results for SMARCA4 in AT/RT-like, AT/RT and MB samples. (C) Both INI1(+) AT/RT-like tumor and INI(−) AT/RT possess distinct mRNA profiles compared with those of PNETs. MDS plots were drawn based on genes that can differentiate AT/RT from AT/RT-like tumors (with a positive false discovery rate (pFDR) threshold of q < 0.001) show the relationships among AT/RTs, PNETs, and INI1(+) AT/RT-like tumors. [file 12920_2015_103_MOESM1_ESM.tiff]
